# Prospective Efficacy and Safety Study of an Innovative Kerascalp Hair Growth Serum in Mild-to-Moderate Alopecia in India: Regrowth Study

**DOI:** 10.7759/cureus.38742

**Published:** 2023-05-08

**Authors:** Malavika Kohli, Anil Ganjoo, Aseem Sharma, Chetan Y Patil, Simran Sethi, Bhagirath Patel

**Affiliations:** 1 Dermatology, Jaslok Hospital, Mumbai, IND; 2 Dermatology and Laser Surgery, Skinnovation Clinics, New Delhi, IND; 3 Dermatology, Skin Saga Centre for Dermatology, Mumbai, IND; 4 Medicine, A. Menarini India Pvt Ltd., Mumbai, IND; 5 General Practice, Cliantha Research, Ahmedabad, IND; 6 Dermatology, Cliantha Research, Ahmedabad, IND

**Keywords:** hair fall, anagen telogen ratio, kerascalp hair serum, pattern baldness, hair loss, androgenic alopecia

## Abstract

Background and aim: Male and female pattern baldness, commonly known as androgenetic alopecia is the most common type of alopecia, often predetermined genetically, which generally affects the scalp and is characterized by progressive terminal hair loss, known as miniaturization. This study aimed to assess the safety and efficacy of Kerascalp hair serum, a unique combination of esculin, ximenynic acid, and lauric acid, extracted from natural sources in subjects with mild to moderate androgenetic alopecia.

Methods: An open-label, single-arm clinical study was conducted on healthy males and females aged 18-60 years. Each subject applied the hair serum once daily for 90 days. The efficacy of hair serum was evaluated in terms of the following outcome variables: anagen and telogen ratio (A:T ratio), hair thickness, hair density, hair fall, and hair strength assessment. Subjects were assessed on day 0, day 30, day 60, day 90, and on follow-up day 120.

Results: Thirty subjects completed all assessment visits. After using the hair serum for 90 days, statistically significant (p<0.0001) improvement was observed in A:T ratio, hair density, hair thickness, and hair strength; a statistically significant reduction (p<0.0001) in hair fall was also observed. Moreover, improvement in general appearance of hair (in terms of hair volume and density) and scalp (in terms of itchiness, redness, roughness, and dryness) was recorded through dermatological assessment at each treatment visit and at follow-up visit compared to baseline. No adverse event was recorded during the study period, and on follow-up.

Conclusions: The results of this clinical study suggest that a 90 days treatment with a phyto-ingredient-based Kerascalp hair serum is safe and effective in significantly improving A:T ratio, hair density, hair thickness, and hair strength, while reducing hair shedding. The improvement in the test parameters persists, even 30 days after stopping the usage of the serum.

## Introduction

Alopecia, the most common condition causing hair loss from the scalp, is a known cause of distress in people. Androgenic alopecia, alopecia areata, and alopecia induced due to chemotherapy are the three major types of non-scarring alopecia among the different types of alopecia which are classified on the basis of symptoms, patterns, and causes [1]. Androgenetic alopecia (AGA), the most common form of baldness affecting both males and females, is characterized by progressive hair loss in a patterned distribution [2]. The main cause of hair loss in males is male pattern baldness (typically occurring any time post-puberty) and in females is female pattern hair loss. The occurrence of this condition is almost same for both males and females, but what differs is the pattern of inheritance. In males, mid-frontal scalp, vertex, and temporal regions are usually affected by hair loss; in females, more thinning of hair is observed on the frontal/parietal scalp along with greater density over occipital scalp [3].

Patterned hair loss is frequently predetermined by genetics. Androgen receptors, both number and affinity, circulating androgens, and hormone metabolism are also known to play an important role in pattern baldness. The metabolism of testosterone leads to production of dihydrotestosterone (DHT) which has a higher binding affinity than testosterone. This results in comparatively faster acceleration of baldness by reducing hair follicles of scalp in a particular pattern in AGA. In the prevention and treatment of AGA, reduction in the level of DHT plays an important role [4]. The changes in the hair cycle dynamics, i.e., gradual decrease in anagen phase period and increase in telogen phase period lead to miniaturization of hairs and finally thinning and balding [5,6].

Prevalence of AGA in Caucasian males is considered to be the highest. The occurrence rates in Caucasian men are estimated to be around 30% in their 30s, 40% in their 40s, and 50% in their 50s. In the Indian population, a study conducted with 1005 subjects showed that in males aged 30-50 years the occurrence of AGA is 58% [7]. Another study conducted on 2476 Indian men has shown that type II is the most common pattern of AGA in 20-59 years age group. Type III vertex is the common pattern of AGA in 60-69 years age group. The pattern of AGA progresses from type I to type VII with increasing age [8]. The other study has shown that types II and III are commonly observed in AGA [9]. In a study conducted on Indian females, the majority of females had Ludwig grade I, followed by grade II and then grade III [10]. In both men and women, studies have demonstrated that the incidence of AGA gradually increases with age [11].

Strong, dense, and healthy hair is usually correlated with beauty, health, and success, especially in today’s day and age. Thus, men and women with AGA are often psychologically distressed. Pattern baldness is known to have a severe impact on social and emotional stigma of the person suffering from this condition [12]. It is also known to have a negative effect on one’s psychosocial state and increases the rate of depression and anxiety. Hence, early treatment of alopecia is of utmost importance. The current standard of treatment for AGA includes topical minoxidil and finasteride. Additional therapies including dutasteride, ketoconazole, hormonal therapy, and naturally sourced products or their extracts for preventing hair loss have also been used. Surgical procedures including minimal invasive procedures like platelet-rich plasma (PRP), low-level laser therapy (LLLT), and micro-needling have become popular in recent years, and hair transplantation surgeries are generally reserved for refractory patients. However, due to the varying efficacy, frequent side effects including the invasiveness of the surgical procedure and poor patient adherence to treatment mostly due to side effects and treatment duration, there remains the need for additional treatments and formulations promoting hair regrowth [13-15]. Therefore, there are constant efforts are being taken in researching newer formulations for effective hair loss treatments to stop the further thinning of hair and to stimulate the regrowth of hair.

Kerascalp hair serum, the investigational product of this study is a unique combination of esculin, ximenynic acid, and lauric acid, extracted from natural sources. Esculin, a coumarin glucoside that naturally occurs in the *Aesculus hippocastanum* (horse chestnut), is purported to work on arterial microcirculation. Esculin improves microcirculation by improving capillary permeability and fragility and also reduces oxidative stress. Ximenynic acid, a di-unsaturated fatty acid, is found in many plant species such as *Ximenia africana *(wild plum) and *Santalum album *(Indian sandalwood). Ximenynic acid is considered a precursor or stimulator of the biosynthesis of eicosanoids which endows the microvascular kinetic activity at the level of small arteries [16]. Lauric acid, saturated fatty acid extracted from natural substances, has a high affinity for hair proteins, and because of its low molecular weight and a straight linear chain structure, it is able to penetrate inside the hair shaft, thereby exerting a protective effect on the damaged hair. In vitro studies have also demonstrated the inhibitory action of lauric acid on 5-α-reductase thus reducing the activity of DHT, addressing the principal pathophysiology of AGA [16-18].

The present study was designed to evaluate the efficacy and safety of Kerascalp hair serum (by A. Menarini India Private Limited), a unique triple combination of esculin, ximenynic acid, and lauric acid, in terms of improvement in anagen and telogen ratio (A:T ratio), hair thickness and density, hair strength along with general appearance of scalp, hair, and skin and reduction in hair fall in subjects with mild to moderate alopecia.

## Materials and methods

Study design and participants

An open-label, single-arm clinical study was conducted to evaluate the efficacy and safety of Kerascalp hair serum in healthy adult subjects with mild to moderate alopecia. The sample size was calculated based on a previous experience of similar study in consultation with the investigator.

**Figure 1:**
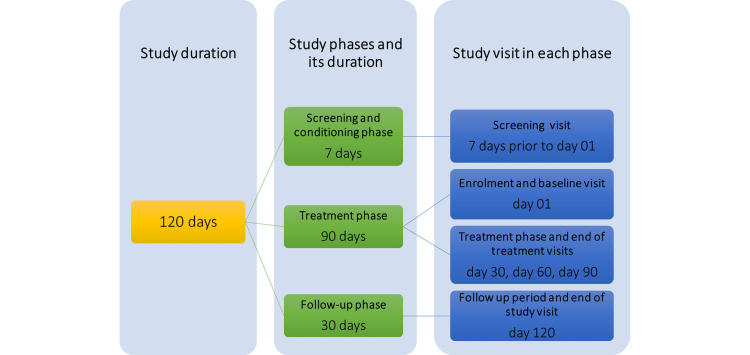
Study duration and visits. Visits for shaving of the test area were scheduled on day 2, day 27, day 87, and day 117.

Dermatological evaluation, instrumental evaluation, and questionnaires were performed for the evaluation of product efficacy. Digital photographs were taken for representative purposes. Safety of the product was assessed by monitoring the adverse events throughout the study duration (Figure 1). The potential subjects were screened based on the inclusion and exclusion criteria prior to baseline assessments. The eligible subjects were given test products for 90 days of application.

Inclusion criteria

Healthy non-pregnant/non-lactating female and male subjects of 18-60 years age group with mild to moderate AGA (males - Norwood Pattern II to IV, and females - Ludwig Scale I to II) were enrolled in this study. Female subjects of childbearing potential must have a negative urine pregnancy test on screening visits. Subjects who were generally in good health and who underwent a blood test for hemogram and a thyroid panel to rule out any abnormalities which could have affected the treatment were included. Subjects willing to refrain from any other treatment for the main indications for which the study test products were being given during the course of the study; subjects who agreed to not use medicated/prescription shampoos/hair care products (containing Minoxidil/anti-hair fall agents) or any other hair fall treatment/hair products other than the test product for the entire study duration; subjects willing to use test product throughout the study period as instructed; subjects willing and able to follow the study protocol to participate in the study and subjects willing to allow photographs of affected areas to be taken during the study duration, were also included in the study.

Exclusion criteria

Subjects fulfilling any of the following listed criteria were excluded from the current study: history of any dermatological scalp condition other than hair loss; previous use of scalp hair growth treatment, hair growth procedures like PRP, hair transplant or laser etc., subjects who had used topical treatment for hair loss, any systemic treatment for at least 90 days before their study participation; chronic oral steroids 90 days before product application and during the study duration; history of substance abuse/addition; subjects who had plans of shaving of scalp hair during the study period; subjects having history/present condition of visibly inflamed or irritated scalp or severe scalp disease, an allergic response to any ingredient of study product; subjects suffering for hair loss due to chemotherapy/autoimmune disorder/immunosuppressive drugs/polycystic ovary syndrome (PCOS)/any major illness etc.; any other condition, as per the discretion of the dermatologist/investigator, which could justify exclusion from the study.

Test product

The test product “Kerascalp” hair serum (by A. Menarini India Private Limited), a unique triple combination of esculin, ximenynic acid, and lauric acid, was provided to all 36 enrolled subjects for application for 90 days. The subjects were asked to apply approximately 2 mL of hair serum once daily on the affected areas on dry hair and scalp and gently massage the product with their fingertips. They were asked to wash their hands with soap after applying hair serum.

Primary efficacy endpoints

The primary efficacy endpoint was assessment of A:T ratio at baseline, i.e., before test product application, at subsequent time points post-product application and at the end of follow-up visit. A:T ratio was assessed by a hair pluck test and microscopic evaluation. This test provides the number and the proportion of hairs in the different phases of the hair cycle.

Secondary efficacy endpoints

Secondary efficacy endpoints include assessment of hair thickness, hair density, hair fall reduction and hair strength at various study follow-up visits compare to baseline visits. Analysis of the hair thickness and hair density was performed using CASLITE Nova (Navi Mumbai, India: Catseye System and Solution). Assessment of hair fall was done by 60-second hair comb test which is aimed to find out range of shedding hair during the combing period. Assessment of hair strength was done by hair pull test which is based on the concept of "gentle" pulling of the hair to bring about shedding of telogen hairs.

Other subjective assessments, such as general appearance of scalp hair and skin, subject’s self-assessment questionnaire for product efficacy, and physician global esthetic improvement scales (PGAIS) at various study follow-up visits compared to baseline visits were also noted. Assessment of general appearance of hair was performed for hair volume (full - medium - small) and hair density (dense - thinned/shed) by dermatologist, while that of scalp skin was performed for redness, roughness, scaliness, and itchiness.

Safety endpoints

Subjects were monitored for adverse events (AEs) at the time of every study visit. They were also requested to contact the investigator to inform them of any incidences of undesirable/adverse events at any point of time.

Statistical analysis

All statistical tests of the recorded data were done using SAS statistical software (version: 9.4; Cary, NC: SAS Institute Inc.). Continuous variables were summarized using tables of descriptive statistics (i.e., mean, standard deviation, median, minimum, and maximum). Categorical variables were summarized using counts and percentages. For continuous variables, the within-treatment analyses were conducted to compare baseline to post-treatment data using paired t-test. For categorical variables, the within-treatment analysis was conducted to compare baseline to post-treatment analysis using Wilcoxon signed-rank test. All statistical tests of hypothesis were done at a 0.05 level of significance.

## Results

Subject disposition and demography

A total of 107 subjects were screened, out of which 36 subjects fulfilling all inclusion criteria, with average (±SD) age of 42.0 (±8.49) years were enrolled. Out of 36 enrolled subjects, five subjects lost to follow-up after repetitive contact attempts and one male subject withdrew consent and hence discontinued from the study. A total of 30 subjects (10 females and 20 males) completed all study visits as per the study design (Table 1).

**Table 1:** Summary of demographic characteristics. N=number of subjects

Age (years), N=36	Treatment group
Mean±SD	42.0±8.49
Median	42.0
Min, max	19, 59

Primary efficacy parameters

**Figure 2:**
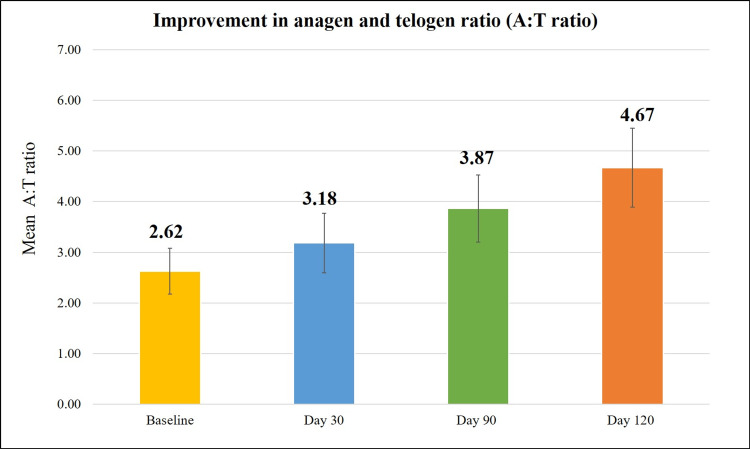
Improvement in anagen and telogen ratio (A:T ratio).

Efficacy of Kerascalp hair serum was measured in terms of A:T ratio and showed significant improvement in A:T ratio at day 90 compared to baseline (51.67%, p<0.0001). Also, after stopping the product usage, significant improvement in A:T ratio (81.42% vs baseline; p<0.0001) was observed, in continuum compared to baseline (Table 2 and Figure 2).

**Table 2:** Percentage change in different hair parameters at each study visit.

Visits	A:T ratio	Hair thickness (μm)	Hair density (number of hairs/cm^2^)	Hair fall reduction with bulb (% change from baseline)	Hair fall reduction without bulb (% change from baseline)	Hair strength (% change from baseline)
Day 30	21.81	3.8	9.9	18.0	20.3	18.5
Day 60	-	7.7	19.7	23.7	25.6	35.1
Day 90	51.67	11.4	29.3	49.8	56.3	42.9
Day 120	81.42	15.7	37.8	77.0	76.1	44.4

Secondary efficacy parameters

**Figure 3:**
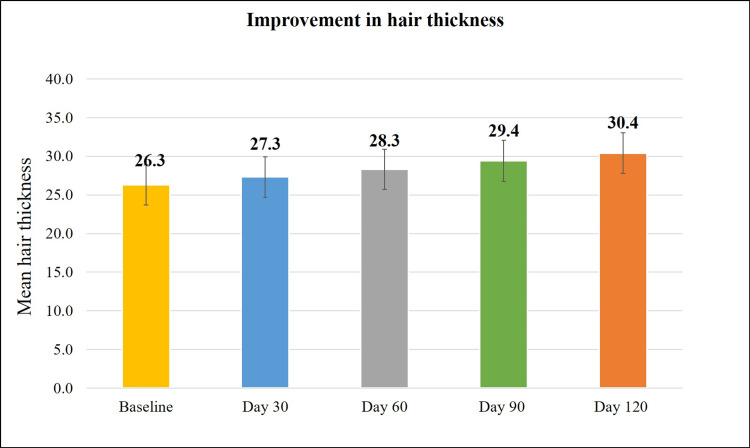
Improvement in hair thickness (in μm).

**Figure 4:**
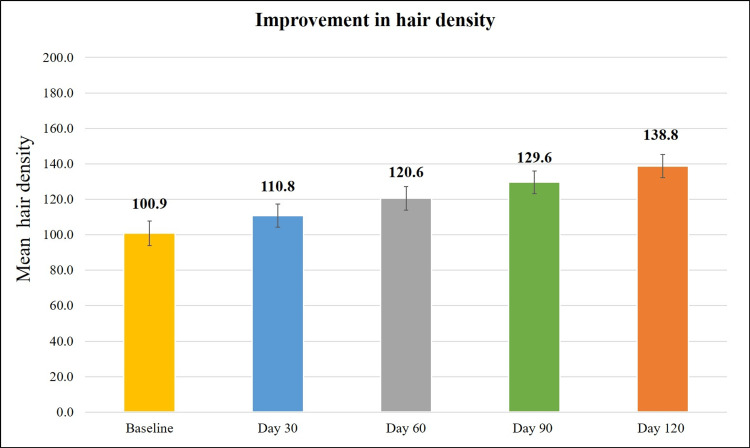
Improvement in hair density. Hair density=number of hairs/cm^2^

**Figure 5:**
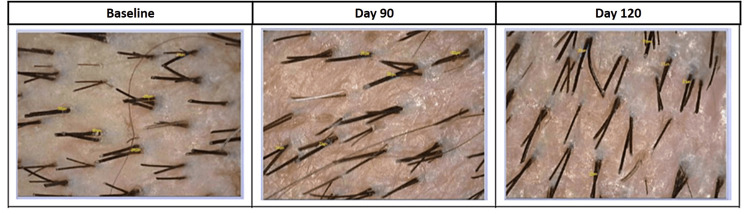
Hair thickness measured by CASLITE Nova after treatment with hair serum and after stopping the product usage. CASLITE Nova (Navi Mumbai, India: Catseye System and Solution)

**Figure 6:**
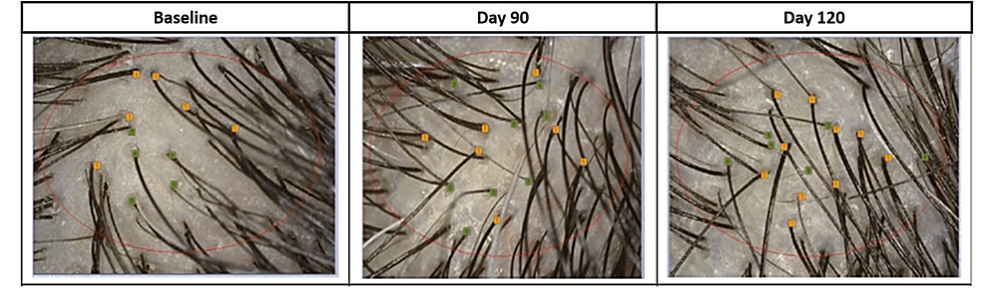
Hair density measured by CASLITE Nova after treatment with hair serum and after stopping the product usage. CASLITE Nova (Navi Mumbai, India: Catseye System and Solution)

**Figure 7:**
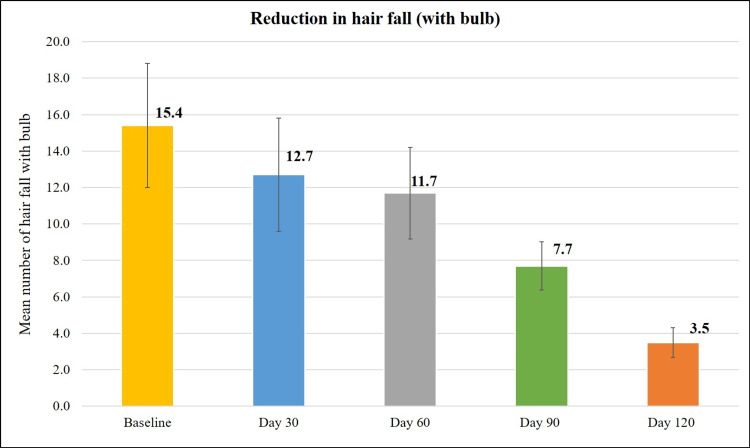
Reduction in hair fall (with bulb).

**Figure 8:**
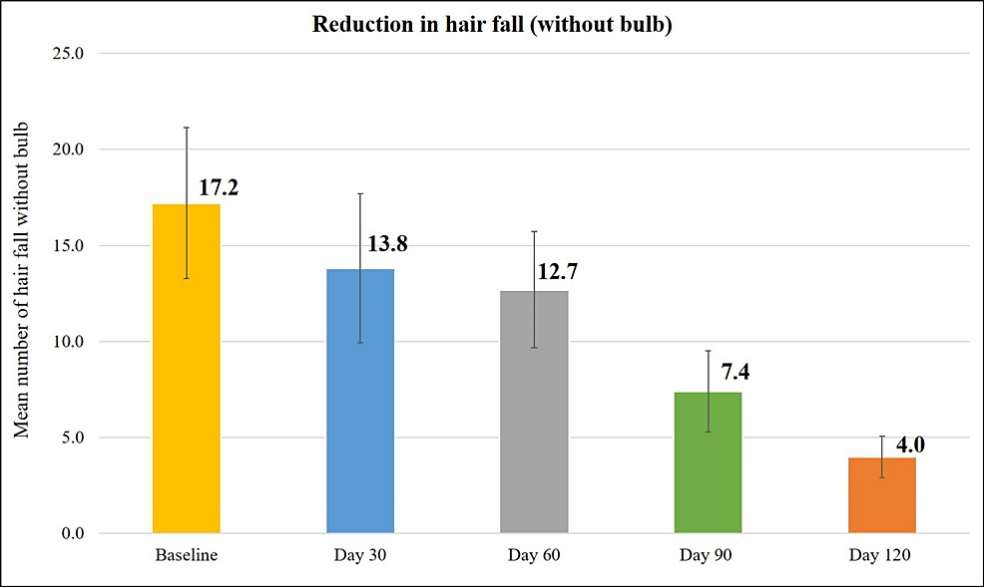
Reduction in hair fall (without bulb).

Treatment with the Kerascalp hair serum resulted in significant improvement (p<0.0001) in secondary efficacy parameter at each study visit compared to baseline (Table 2) like hair thickness and hair density (Figures 3-6) and hair fall (with bulb and without bulb) (Figures 7, 8).

**Figure 9:**
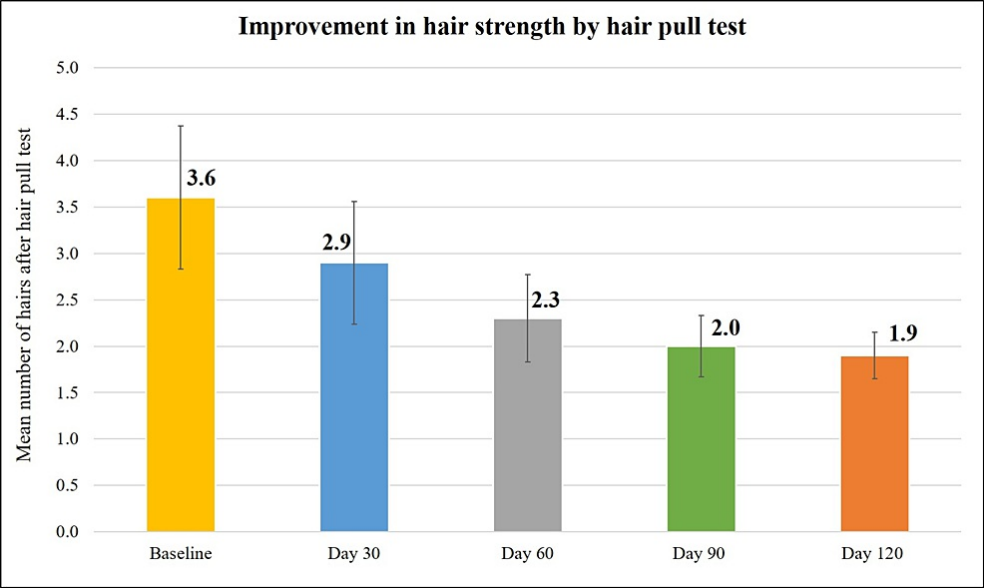
Improvement in hair strength.

Treatment with the Kerascalp hair serum also resulted in significant reduction (p<0.0001) in number of hair shafts after hair pull test at each follow-up visit compared to baseline (Table 2 and Figure 9).

General appearance of scalp hair and skin

**Figure 10:**
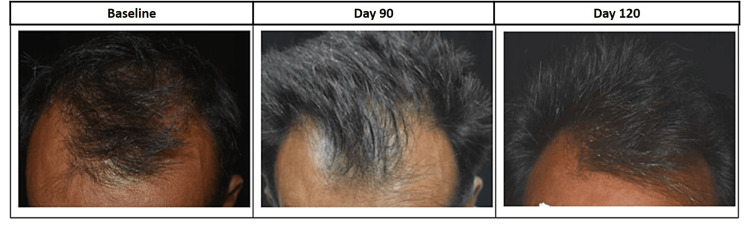
Global photographs after treatment with hair serum and after stopping the product usage.

General appearance of scalp hair in terms of hair density and hair volume shows that after application of Kerascalp hair serum for 90 days, 89.29% subjects showed improvement in hair density from thin to dense hair and 100% subjects showed improvement in hair volume from less to medium to a fuller hair volume. Also, after stopping the product usage, 93.33% subjects showed improvement in hair density from thin to dense hair and 100% subjects showed improvement in hair volume from less to medium to fuller hair volume (Figure 10). General appearance of scalp skin in terms of itchiness, redness, roughness, and dryness shows that after application of Kerascalp hair serum for 90 days, no subject had scalp itchiness and showed improvement in scalp redness, roughness, and dryness at day 90 and day 120.

Subject self-assessment

All the subjects were in agreement that the Kerascalp hair serum reduces hair shedding, makes hair appear dense and thick, and makes hair feel soft and smooth; 96.43% subjects were in agreement that the test product makes bald spots and thin areas appear smaller and lesser; they did not feel any hypersensitivity.

Physician global esthetic improvement scales (PGAIS) and safety assessment

After the application of Kerascalp hair serum for 90 days, 96.43% subjects showed much/marked improvement and 3.57% subjects showed obvious improvement in appearance from the initial condition. Also, after stopping the product usage, 53.33% subjects showed very much improvement/optical cosmetic result, 43.33% subjects showed much/marked improvement and 3.33% subjects showed obvious improvement in appearance from the initial condition. No apparent adverse events were observed.

## Discussion

Even though AGA is common hair condition faced by a large population, it can be one of the most difficult conditions to tackle; reasons being the selection of treatment which often involves consideration of important factors like products’ effectiveness, possibility of side effects, and the protracted cost of treatment [19].

There are many treatments available for alopecia in adults which primarily include topical minoxidil and finasteride. The mode of administration includes topical application, oral intake, and injections [20]. Many of these treatments can also cause adverse effects. The most commonly observed side effect of minoxidil is pruritus on the application site. Although less common, contact dermatitis can also occur [21]. The use of minoxidil in women may cause facial hypertrichosis, and the therapy is classified as pregnancy category C [22]. Since minoxidil is required to be used frequently, compliance becomes a critical factor when considering its usage [23]. If the product usage is terminated before its intended treatment period, progressive hair loss can be expected within 12-24 weeks [24]. While oral finasteride is also USFDA-approved, it is not devoid of side effects. Among the reported ones are gynecomastia, testicular pain, hypersensitivity reactions, impaired sexual function in males, and contraindicated in pregnant women and women of childbearing potential because of the risk of feminization of a male fetus [2]. Since finasteride is required to be used chronically, compliance can become a critical factor when considering its usage [19]. Finasteride has shown to be effective in treating AGA and its long-term use has also shown improvement in hair growth along with permanent stabilization of hair loss [25,26]. Side effects from finasteride use include orthostatic hypotension, dizziness, and impaired sexual function in males all of which may or may not decrease with time [27]. Oral dutasteride is known to be more efficacious in blocking DHT thereby promoting hair growth as compared to finasteride. Despite being superior to finasteride, finasteride is still likely to be prescribed in treating AGA [19]. Similar to finasteride, the side effects of oral dutasteride include impaired sexual function in males [28].

Therefore, there is an increased, recent interest in using naturally sourced products or their extracts for preventing hair loss. Various plant-based ingredients like procapil, baicapil, redensyl, capixyl, ginkgo biloba, caffeine, sinapic acid, and shikimic acid have been shown to promote hair growth and treat AGA. Procapil, a combination of vitamin-rich matrikine with apigenin and oleanolic acid from olive tree leaves, primarily targets poor scalp microcirculation, follicle aging, and follicle atrophy caused by DHT. Baicapil, a synergistic combination of *Scutellaria baicalensis*, soy, and wheat sprouts, stimulates hair growth, increases hair density, and reduces hair loss. *Ginkgo biloba*, a tree native to China, is an effective hair loss treatment that helps to support the healing of weak hair follicles. For the existing natural treatments, the different modes of action reported include DHT and 5-α-reductase blockers, nutritional support (vitamins, minerals, antioxidants), and an increase in blood circulation in the scalp. The role of DHT in hair fall is well-established scientifically, and the plant extracts with these nutritive components, essential oil, and DHT-blocking activity have shown to have great potential in treating alopecia and hair loss [13].

Apart from the above treatment options, other alternative techniques are available in treating AGA which include surgery, platelet-rich plasma (PRP), low-level laser therapy (LLLT), and microneedling. Hair restoration surgery comprises either scalp reduction surgery or hair transplantation or a combination of both. Hair transplantation is usually reserved for severe grade of alopecia. Platelet-rich plasma (PRP) is also an alternative treatment for AGA, which is indicated for early-stage AGA where hair follicles are intact and more significant hair restorative efficacy can be observed. While PRP injections are a safe option if given by trained medical personnel, these treatments are not advisable for everyone, especially for those with a history of bleeding disorders or those who fear injections. Low-level laser therapy (LLLT) is a more commercially viable method for treating AGA which is usually administered through home-use devices in the form of combs, helmets, and caps. Side effects like mild paresthesia, such as burning sensation, dry skin, headache, and pruritus, can be observed on using LLLT [19]. Studies have shown that microneedling seems like a safe and effective adjuvant therapy and can boost the penetration of topical therapies [19]. Common side effects include bruising, pain, and folliculitis. Compliance might an issue due to the cost of the treatment and it being painful [29].

Nowadays, an increasing demand for natural and plant-based treatments to treat hair loss is palpable. Many therapies which can potentially treat the AGA are available in an alternative, non-conventional, traditional healthcare system. Many plant-based ingredients, estimated to be more than a thousand, have been studied for the hair care benefits they may provide. Some of these like rosemary oil, hibiscus flowers, extract of grape seed, and sage help in improving the blood flow and provide protection from hair fall. Ingredients like ginkgo, green tea extract, and emu oil decrease the level of DHT by inhibiting the action of 5-alpha reductase, thus confirming the use of these plant-based ingredients as a potential natural cure for alopecia [30]. Many studies have been conducted to evaluate the effectiveness of botanical ingredients in treating hair loss. It becomes evident after reviewing the literatures available on earlier studies, that the plant-based ingredients when combined together can be effective in treating alopecia [31,32].

The investigation test product in this study, Kerascalp hair serum, is a unique triple combination of esculin, ximenynic acid, and lauric acid, was evaluated clinically for its potential benefits in androgenic alopecia in Indian subjects. Maramaldi et. al. conducted a placebo-controlled trial in Italy with multicomponent esculoside, ximenoil, and lauric acid hair loss lotion in 40 subjects. At the end of the three-month treatment, in the test group, there were significant improvements in trichogram findings - a significant increase of hair in anagen phase and a significant decrease of hair in telogen and dystrophic anagen phases have been observed. Moreover, the subjective evaluation of the efficacy and the cosmetic parameters of the tested lotion showed an overall positive effect [16].

After using the Kerascalp hair serum for 90 days, significant improvement was observed in each assessment visit in A:T ratio, hair density, hair thickness, and hair strength, while reducing hair fall significantly. The dermatological assessment showed improvement in general appearance of scalp hair and skin throughout the treatment period. Similar results were observed even after stopping product usage. Results of subjective assessment also confirmed the efficacy of test serum in improvement of the hair and scalp condition. None of the subjects had any local intolerance during the study period confirming the safety of the test product and no adverse events were neither reported nor observed during the course of the study. However, this study was limited by non-comparative study design, short follow-up period, and limited sample size, as it was an open-label study with no comparative arm.

## Conclusions

In the present open-label, single-arm clinical study, the phyto-ingredient-based Kerascalp hair serum was evaluated for its safety and effectiveness. The combined effect of this unique triple combination of esculin, ximenynic acid, and lauric acid on regular application once a day on the scalp significantly improves A:T ratio, hair density, hair thickness, and hair strength while reducing hair fall. It also helps in improving general appearance of scalp hair and skin. Similar results were observed even after 30 days of stopping the product usage. Furthermore, no apparent local intolerances and adverse events were reported during the study, thus proving to be an effective option for mild to moderate alopecia. Based on encouraging results from the present study, it can be used as an adjuvant or as monotherapy, depending upon individual patient assessment, in the management of mild to moderate alopecia of the scalp. However, further controlled clinical studies are needed to confirm the findings of this study and to substantiate synergistic action when used as an adjunctive treatment.
